# Patients with hepatic breast cancer metastases demonstrate highly specific profiles of matrix metalloproteinases MMP-2 and MMP-9 after SIRT treatment as compared to other primary and secondary liver tumours

**DOI:** 10.1186/s12885-016-2382-2

**Published:** 2016-06-08

**Authors:** Olga Golubnitschaja, Kristina Yeghiazaryan, Helena Stricker, Daniela Trog, Hans H. Schild, Leonard Berliner

**Affiliations:** Department of Radiology, Rheinische Friedrich-Wilhelms-University of Bonn, Bonn, Germany; radiox Strahlentherapie Hamm, Hamm, Germany; New York Methodist Hospital, NY Presbyterian Healthcare System, Brooklyn, NY USA

**Keywords:** Predictive preventive personalised medicine, Hepatic carcinoma, Breast cancer, Metastasis, SIRT, Biobanking, Blood test, Matrix metalloproteinase patterns, Patient stratification, Prognosis

## Abstract

**Background:**

Patients with primary and metastatic liver malignancies represent a highly heterogeneous patient pool characterised by some of the shortest life expectancies amongst oncology patients. Investigation and better understanding of liver malignancies is an emerging field which requires high-quality multidisciplinary research and collaboration.

**Methods:**

A study of 158 patients with primary hepatic carcinomas and secondary liver metastases, altogether 15 cancer types of different origin, who underwent selective internal radiation therapy (SIRT) with Yttrium^90^ or transarterial chemoembolisation, was undertaken in an effort to detect distinguishing features with respect to activity profiles of both blood matrix metalloproteinase (MMP-2 and MMP-9).

**Results:**

Noteworthy, stratification of all hepatic cancer groups with respect to MMP-2 and MMP-9 activities revealed characteristic patterns specifically in patients with hepatic breast cancer metastases who had undergone SIRT. In contrast to all other groups, these patients demonstrated well-consolidated profiles of both MMPs, reflecting a common feature, namely an immediate and durable increase of their activity after the SIRT treatment. Although the total number of patients in the breast cancer group is relatively small (15 patients), since increased activities of MMP-2 and MMP-9 are well known prognostic factors for poor outcomes of oncologic patients, the significance and clear group-specificity (from 15 ones investigated here) of this previously unanticipated finding requires particular attention and further investigations. Particularly important is to determine, whether this increase of the metalloproteinase activity was provoked by SIRT, as well as whether special selection criteria are required for patients with breast cancer metastases to the liver who are being considered for SIRT.

**Conclusions:**

It is recommended that a more focused, multidisciplinary and large-scaled investigations of the possible adverse effects of SIRT in patients with advanced metastatic disease of breast cancer be undertaken, with an appropriate patients’ stratification, set-up of the relevant patient profiles and disease modelling.

## Background

Patients with primary and metastatic liver malignancies represent a highly heterogeneous patient pool characterised by some of the shortest life expectancies amongst oncology patients. Liver cancer is the sixth most common cancer worldwide, with more than 782,000 new cases diagnosed in 2012 (6 % of the total) [1]. Primary hepatic tumours frequently originate from well-defined local triggers such as organ-specific chronic inflammation, hepatitis and/or liver cirrhosis. Secondary, or metastatic, liver malignancies are even more common than primary tumours, presenting a heterogeneous and complex clinical picture. The most common sites of primary tumour are breast, lung, and colorectal cancer [2]. Some authors have reported hepatic metastases in as many as 40 to 50 % of adult patients with extrahepatic primary tumours.

The approach to diagnosis, prognosis, and treatment is dependent upon the localisation and determination of the biological activity of the original tumour, with wide variation in expected response of liver metastases to treatment. For certain tumours, such as a solitary metastasis to the liver from colorectal cancer, there is a favourable survival rate following liver resection that represents relatively simple mechanisms of a local spread of malignant cells in an immediate neighbour-organ within the abdominal compartment.

A completely different set of circumstances is encountered when there is systemic spread of distant metastases. The process is highly complex and requires certain obligatory steps at the cellular and molecular level: primary tumourigenesis, local tumour-related invasion and angiogenesis, down-regulated cell-cell adhesion within cancerous lesions, systemic spread of cancerous information (CNAPs, cell-free DNA/RNA, circulating tumour cells), creation of organ/tissue specific metastatic environment, metastatic seeding, entry into dormancy, metastatic growth provocation, and, genotype modifications under therapy conditions frequently leading to more aggressive phenotypes of secondary and tertiary tumours compared to the original ones.

In particular, the above listed processes are characteristic for liver metastases from breast cancer. Recent publications in the area demonstrate that despite improved treatments, the prognosis for non-operable patients with liver metastases from breast cancer metastatic disease (BCMD) remains poor [3].

Our current study of BCMD is motivated by the following reasons:

1. BCMD is considered an incurable disease: there are poor outcomes and a low life expectancy of this patient cohort [4].
2. The liver is one of the most frequent sites of involvement in patients with BCMD [5]. Depending on the sub-type of breast cancer and infectious component, the liver involvement may reach 25 % of patients and more [6, 7].
3. In postoperative breast cancer patients liver metastases appear earlier than other distanced metastases [8]; a spontaneous dormancy of metastatic breast cancer cells to the liver has been demonstrated [9].
4. The BCMD to liver is linked to the particularly poor outcomes: current studies with multivariate analysis confirmed liver involvement in BCMD as independent predictor of worse overall survival [10].
5. The molecular background of the therapy resistance in BCMD has not been adequately studied yet. Stratification of patients into prognostic groups is essential.

An extensive degradation of extracellular matrix is the essential attribute of tumour progression and aggressive metastatic disease. Within the protein family of proteases degrading the extracellular matrix, both gelatinases A and B—metalloproteinases MMP-2 and MMP-9, respectively—are well known prognostic factors that, when elevated, are indicators of poor outcomes for oncologic patients in general, and, in particular, promoting liver metastases [11, 12], metastatic disease in the most aggressive breast cancer phenotypes (such as triple-negative breast cancer) [13] and formation specifically of liver metastases in breast cancer [14]. Further, the patterns of activities of both MMP-2 and −9 are regulated by many molecular mechanisms applied via a cascade of individual steps including transcription, translation, several events of post-translational modification and, finally, MMP-activity inhibition by TIMPs. In this comprehensive situation, it is very difficult to expect a linear correlation between expression rates (e.g. measured by ELISA) and levels of activity (Zymography used in the project). From the entire regulation cascade, the “end-products”, namely effective activity levels of both molecular targets have the highest relevance for biological aspects of metastatic disease and practical clinical utility. Consequently, the current paper is focused on specific patterns of activities of both MMP-2 and −9 in patients with primary and metastatic liver malignancies.

## Methods

### Recruitment of patients with primary and secondary hepatic carcinomas and blood sample collection

A total of 158 patients with primary and secondary hepatic carcinomas were recruited at the Department of Radiology, Rheinische Friedrich-Wilhelms-University of Bonn.

#### Including criteria

- primary hepatic carcinoma
- hepatic metastases
- treatment by SIRT (Selective Internal Radiation Therapy)
- treatment by TACE (Transarterial Chemoembolisation)

#### Excluding criteria

- pregnancy
- acute infections (but not chronic hepatitis)
- alcohol abuse
- genetic disorders and disorders with premature ageing (Down Syndrome, Werner Syndrome, Alzheimer’s disease, others)

**Fig. 1:**
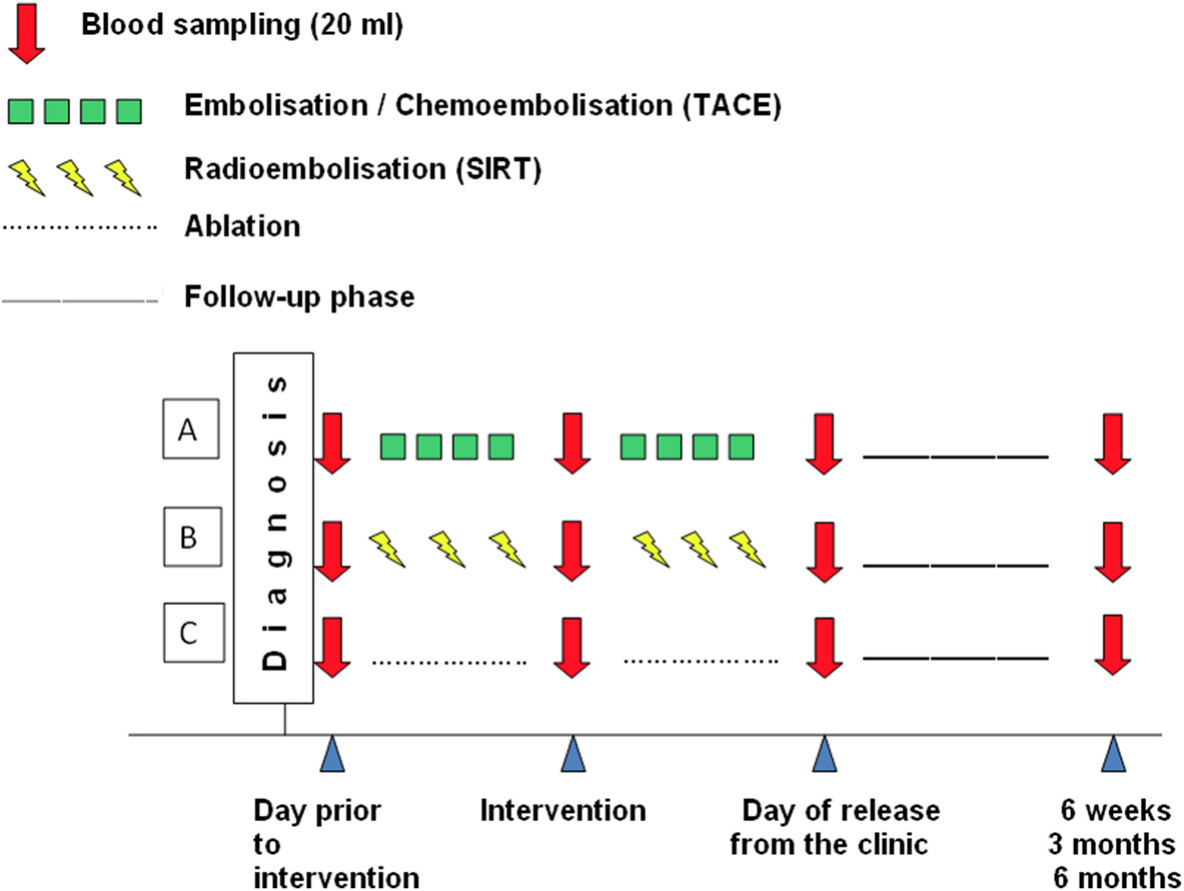
Workflow. The scheme summarises how the entire study has been designed and experimental approach performed in the patients pool

The recruited patients were grouped according to the primary diagnosis (original tumour), gender, and therapy approach chosen and applied to the patient—see the workflow presented in Fig. 1. Blood samples of all patients were taken

- 1st time prior to the intervention
- 2nd time at the day of the release from the clinic
- 3rd, 4th and 5th times during the follow-up phase.

### Blood samples collection and biobanking

Blood samples (20 ml) anti-coagulated with heparin were collected from the patients. For the current study, blood plasma (500 μl) has been separated by short centrifugation and stored at −80 °C until the zymographic analysis were performed as described below.

For the biobanking and all follow-up analyses, circulating leukocytes were separated using Ficoll-Histopaque gradients (Histopaque 1077, Sigma, USA) as described previously [15]. Briefly, blood samples were diluted with equal volumes of physiological buffer solution (PBS, Biochrom AG, Germany). Then, 2 ml of histopaque were placed into 10 ml sterile centrifuge tubes and 5 ml of diluted blood samples were carefully layered onto each histopaque gradient. Gradients were centrifuged at 475 g and 20 °C for 15 min. The leukocytes bands were removed from the interface between plasma and histopaque layers of each tube and collected into one 50 ml tube. The total volume was brought to 50 ml with cold Dulbecco’s Modified Eagle Medium (DMEM, Gibco™, USA). The cell suspension was washed three times with PBS and the total number of cells was determined. Cells were finally resuspended in PBS-DMSO solution, aliquoted into eppendorf tubes and stored at −80 °C until molecular profiling in circulated leukocytes might be needed to be performed.

### Zymography

For determination of gelatinase activity of MMP-2 and MMP-9 in blood serum "Criterion™ Zymogram Gel" (Bio-Rad, USA) were used according to the instructions of the manufacturer. Two microliters from individual serum samples were electrophoresed under non-reducing conditions using Criterion™ Precast Gel System (Bio-Rad, USA). After electrophoresis, each gel was incubated at room temperature in 2 % Triton X-100 for 2 x 30 min in order to remove the traces of sodium dodecyl sulphate, and then incubated overnight at 37 °C in buffer (150 mM NaCl, 50 mM Tris–HCl, pH 7.6, containing 5 mM CaCl_2_ and 0.02 % NaN_3_). Afterwards a staining with 0.5 % Coomassie blue G-250 (Sigma, USA) was performed for each gel. The proteolytic activity of each gelatinase (A and B) was identified as a clear band on a blue background according to the correspondent molecular weight of each gelatinase (A and B that corresponds to the Metallproteinase-2 and −9, respectively). Gels were dried between cellophane sheets with a GelAir™ Drying System (Bio-Rad, USA) and then scanned with a yellow filter using Adobe Photoshop (Adobe System, USA) in grey-scale mode. Densitometric analysis of zymographic lysis zones at 66 and 86 kDa for gelatinases A and B respectively was performed using “Quantity One” imaging system (Bio-Rad, USA).

## Results

### Patient data analysis

Patient data analysis is summarised in the Table 1.

**Table 1:** Summary of the patient data analysis

Primary cancer type	Hepatocellular carcinoma		Colorectal cancer		Breast cancer		Cholangiocellular carcinoma		Neuroendocrine tumour		Pancreatic cancer		Lung carcionoma		Esophageal cancer		Ovarian cancer		Cervical cancer		Gastric cancer		MANEC		Uveal melanoma		Urothelial cancer		Cancer of unknown primary	
Gender	M	F	M	F	M	F	M	F	M	F	M	F	M	F	M	F	M	F	M	F	M	F	M	F	M	F	M	F	M	F
No of Cases	45	14	30	18	-	15	4	6	5	3	2	1	1	2	2	-	-	2	-	1	2	1	-	1	-	1	1	-	1	-
Age (Ø)	67.98	66.79	64.73	61.22	-	55.33	65.25	61.5	55.8	60.67	52.5	50	55	57.5	57	-	-	49.5	-	52	58	61	-	31	-	73	61	-	46	-
TACE (in %)	48.89	42.86	3.33	0	-	0	0	0	0	0	0	100	100	0	0	-	-	0	-	0	0	0	-	0	-	100	0	-	0	-
SIRT (in %)	51.11	57.14	96.67	100	-	100	100	100	100	100	100	0	0	100	100	-	-	100	-	100	100	100	-	100	-	0	100	-	100	-

Patient data analysis of 158 patients with primary hepatic carcinomas and secondary metastasis revealed following differentiations:

- for the gender: 65 females and 93 males

Cancer types: 59 Hepatocellular Carcinoma, 48 Colorectal Cancer, 15 Breast Cancer, 10 Cholangiocellular Carcinoma, 8 Neuroendocrine Tumour, 3 Pancreatic Cancer, 3 Lung Cancer, 2 Esophageal Cancer, 2 Ovarian Cancer, 1 Cervical Cancer, 3 Gastric Cancer, 1 Mixed Adenoneuroendocrine Carcinoma, 1 Urothelial Cancer, 1 Uveal Melanoma, 1 Cancer of Unknown Primary

- Therapy: 79.75 % SIRT and 20.25 % TACE

### Mortality in the cohort of the patients with hepatic breast cancer metastases

The cohort of the patients with hepatic breast cancer metastases for 100 % was treated with the SIRT. The mortality rates were recorded as following:

- one third part of the group died within 3 months after the treatment
- 60 % died within 1 year.

### MMP-9 patterns in blood

#### A. All primary and secondary hepatic carcinomas stratified according to individual MMP-9 profiles in blood

**Fig. 2:**
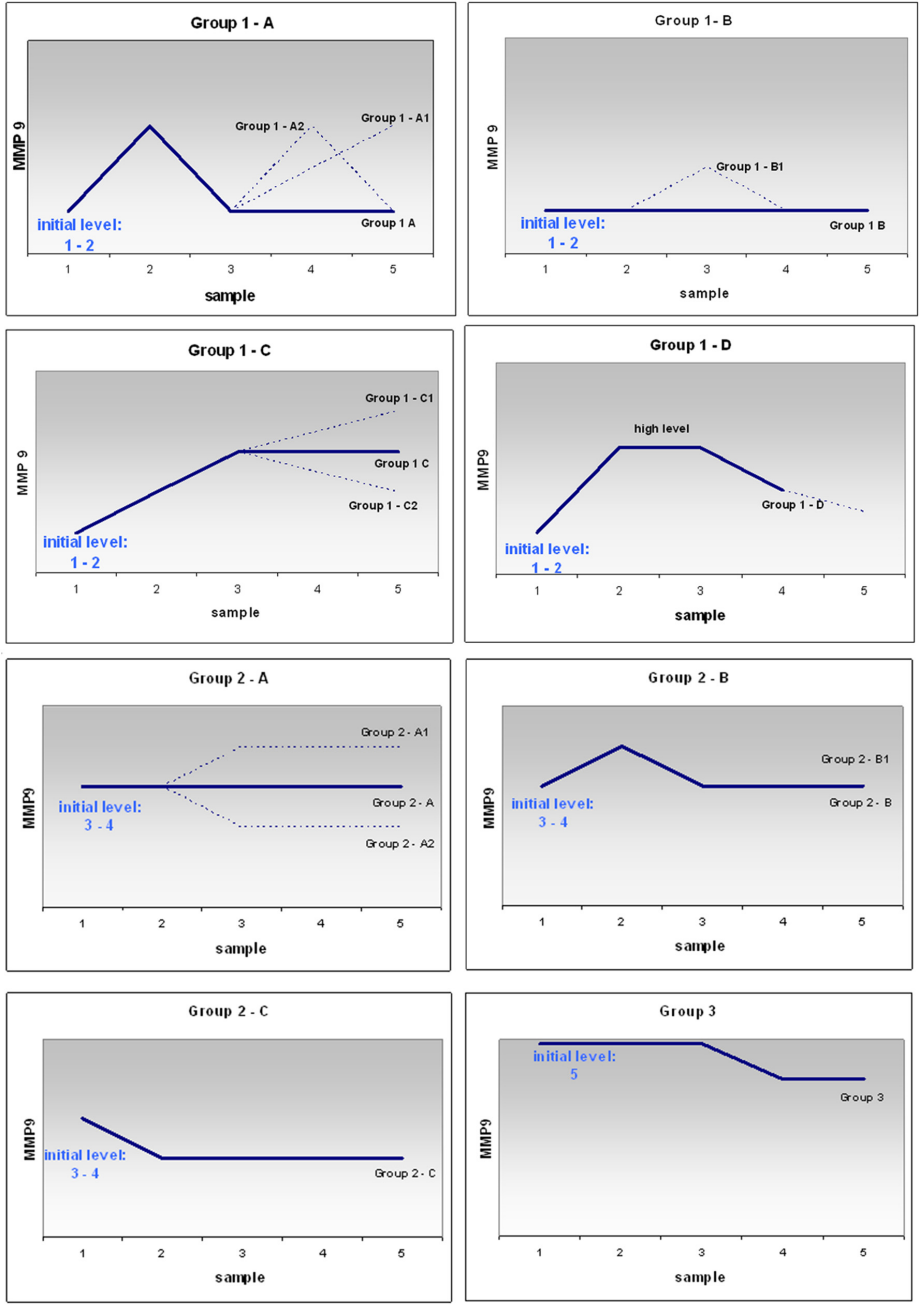
MMP-9 patterns. Above constructed patterns have been monitored for activities of MMP-9 in blood serum of individual subgroups of all hepatic carcinoma patents investigated in the current study

Stratification of all 158 hepatic carcinoma patients considering individual MMP-9 profiles in blood revealed 8 key patterns as summarised in Fig. 2. In general, the patterns’ distribution across the individual groups of patients stratified according to the gender, cancer, and therapy type was highly heterogeneous.

#### B. Cohort of the patients with hepatic breast cancer metastases

In contrast to all other groups, the patients with hepatic breast cancer metastases demonstrated well consolidated MMP-9 profiles reflecting the main common feature, namely an immediate increase and stably high level of the MMP-9 activity after the SIRT application that corresponds to the patterns 1A, 1C, 1D, 2B1. The four patterns have been recorded as the characteristic for this patient cohort.

### MMP-2 patterns in blood

#### A. All primary and secondary hepatic carcinomas stratified according to individual MMP-2 profiles in blood

**Fig. 3:**
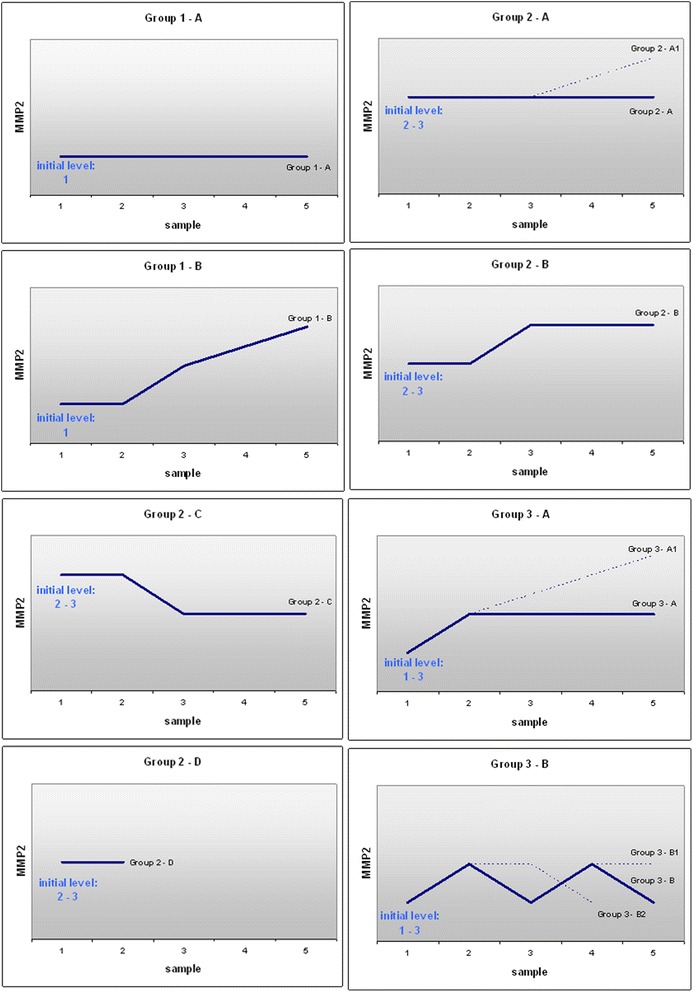
MMP-2 patterns. Above constructed patterns have been monitored for activities of MMP-2 in blood serum of individual subgroups of all hepatic carcinoma patents investigated in the current study

Stratification of all 158 hepatic carcinoma patients considering individual MMP-2 profiles in blood revealed 8 key-patterns as summarised in Fig. 3. In general, the patterns’ distribution across the individual groups of patients stratified according to the gender, cancer and therapy type was highly heterogeneous.

#### B. Cohort of the patients with hepatic breast cancer metastases

In contrast to all other groups, but similarly to the MMP-9 patterns, the patients with hepatic breast cancer metastases demonstrated also well consolidated MMP-2 profiles reflecting the same main feature, namely an increasing and stably high level of MMP-2 activity after the SIRT application that corresponds to the patterns 2A1, 2B, 3A, 3B1. The four patterns have been registered as the characteristic for this patient cohort. A very few exceptions demonstrated the pattern 2D (unchanged after the therapy); however, the initial level of the MMP-2 activity in those cases was extremely high.

## Discussion

For selected patients with primary or metastatic hepatic cancer, liver resection is felt to offer the best overall chance of cure. Unfortunately, at the time of the diagnosis only 10–20 % of cases are candidates for a surgical approach. Consequently, the overwhelming majority of the patients with hepatic tumours are treated with standardised palliative approaches which aim at stabilising the tumour’s growth and slowing down its metastatic activity. The success rates of these treatments are widely variable, resulting in survival rates that range from several weeks to over two years following treatments. In particular, it is unclear why tumours with apparently similar anatomic characteristics, undergoing similar treatment regimens, lead to different individual outcomes. The matter is hardly explainable by currently available diagnostic and prognostic tools. An approach to achieving greater understanding of the wide variation in biologic activity and patient-specific response to therapy utilising an Information Technology framework, with respect to hepatocellular cancer, has been recently proposed [16, 17]. Work is currently underway in understanding variability in individual responses through data-mining utilising graph theory and Bayesian inference.

In addition, variations in patient response to treatment have motivated the authors to build a multidisciplinary consortium to analyse the diversity of liver malignancies, to develop multilevel studies, and to seek potential explanations for discrepancies recorded for the treatment outcomes. Accordingly, in the study presented herein, 158 patients with liver malignancies of 15 different types of the cancer origin were treated with TACE or SIRT, and their outcomes were analysed. A small but striking subset of subjects, namely the patient cohort with hepatic breast cancer metastases treated by SIRT displayed group-specific remarkable findings:

1. the mortality rates were high: one-third of the group died within 3 months after the treatment and 60 % died within 1 year;
2. in contrast to all other groups, the patients with hepatic breast cancer metastases demonstrated well consolidated MMP-9 and MMP-2 profiles, reflecting a unique, common feature, namely an immediate and permanent follow-up increase in the activity rates of both matrix metalloproteinases after the SIRT treatment;
3. this patient cohort demonstrated highly characteristic molecular patterns, in contrast to all other sub-groups.

The above summarised results are well in consensus with statistics, observations and conclusions made by some recently published studies. The BCMD to liver is linked to the particularly poor outcomes: current studies with multivariate analysis confirmed liver involvement in BCMD as independent predictor of worse overall survival [10]. In postoperative breast cancer patients liver metastases appear earlier than other distanced metastases [8]. Furthermore, a spontaneous dormancy of metastatic breast cancer cells to the liver has been demonstrated [9].

It is necessary to mention that there have been several publications reporting favourable results with SIRT for BCMD to the liver. Hence, Coldwell et al. reported on 34 women with unresectable breast cancer metastatic to the liver treated with SIR-Spheres. The selection criteria included only those patients with an ECOG performance score of 0 or 1 with an expected survival of at least 3 months. There was a complete response in 17 % of evaluable patients by PET imaging at 12 weeks, a partial response in 58 %, stable disease in 20 %, and disease progression in 5 %. 36 of 44 patients (86 %) were alive at 14 months [18]. Jakobs et al. reported in a study of 30 patients that there was a partial response in 61 % of evaluable patients, with stable disease or minor response in 35 %, and disease progression in 4 %. The median overall survival was 11.7 months [19]. Cianni et al. reported on 49 patients with breast cancer liver metastases who had failed prior chemotherapy and were treated using SIR-Spheres microspheres. By CT and PET criteria, there was a complete or partial response in 49 % of evaluable patients, with stable disease in 35 % and disease progression in 16 %; technical success rate and effectiveness estimated at 3 months were respectively of 98 % and 80 %; median progression-free survival and overall survival were 9.2 and 11.6 months, respectively [20]. However, current literature does not establish clearly, who may undergo SIRT for breast cancer metastases to the liver.

A more focused and multidisciplinary study in the future, with a suitable number of subject and control patients, will be necessary to determine the possible adverse effects of SIRT. The study should be designed with proper stratification with respect to each patient’s performance status, prior to the therapy, extent of metastatic disease, and liver function.

We also know that hepatic embolisation, chemotherapy, and radiation therapy create changes at the molecular and cellular level, including changes involving extra-cellular structures (i.e. the microenvironment) in, as yet, poorly understood and potentially profound ways. This includes stimulation of angiogenesis due to hypoxia that stimulates angiogenesis and, therefore, tumour growth, and now, the possibility of increased matrix metalloproteins. Large scale studies on human subjects are limited with respect to these phenomena. Korse et al. found a temporary elevation in angiogenic growth factors (vascular endothelial growth factor (VEGF), endothelin-1 (ET-1) and C-terminal proendothelin-1 (proET-1)) in the blood in twelve patients with well-differentiated neuroendocrine tumours and liver metastases who had undergone hepatic artery embolisation [21]. Whereas elevation of MMPs were found to be associated with SIRT in the study reported herein, Daniele et al. reported that 75 patients with HCC showed statistically significant reduction in MMP-2 following treatment with TACE [22].

There is growing evidence that tumour irradiation may induce a variety of responses that might produce tumour radioresistance, growth, and recurrence. While radiation-induced DNA damage, vascular damage, and radiation–specific fibrosis serve to favour tumour regression, certain effects of radiation have been shown to have properties that may result in undesirable effects. While a review of the overall effects of radiotherapy on tumour biology relating to the microenvironment, immune response, resistance to hypoxia, and rapid growth is beyond the scope of this paper, a few points are of interest.

Cancer-associated fibroblasts (CAFs) constitute the majority of cells within the stroma in many carcinomas. These cells actively interact with neoplastic cells and form a myofibroblastic microenvironment that promotes cancer growth and survival, and supports malignancy. They participate in the remodelling of peritumoural stroma, which is a prerequisite of neoplastic cell invasion, expansion, and metastasis [23]. CAFs are known to secrete extra-cellular matrix proteins (such as tenascin C and collagen I), cytokines (such as hepatocyte growth factor, platelet-derived growth factor and chemokine ligand 12) and matrix remodelling enzymes (such as matrix metalloproteinases—MMPs) [24]. Unlike myofibroblasts that arise in response to inflammation or wound healing, CAFs may be resistant to apoptosis and irreversibly activated by radiation. The heterogeneous nature of CAFs varies according to tumour type and the stage of disease progression, and may also determine whether they exhibit tumour-promoting or tumour-inhibiting roles [24]. The effects on CAFs by radiotherapy have not been studied in detail at this time. However, there is evidence that radiotherapy may play a role in tumour survival at the level of the extra-cellular matrix through its effect on the transmebrane receptor, β1 integrin, and that that β1 integrin signalling in pancreatic cancer cells is required for stromal-mediated radioprotection. By analogy, investigation of the specific effects of radiation on the production and action of MMPs is warranted, in view of the findings reported herein.

Despite the fact that all 158 patients, with 15 forms of liver malignancies, were subjected to either TACE or SIRT, it was only in the breast cancer group that a unique and significant experimental finding was detected. The authors suggest that this emphasises the importance of the origin of a primary tumour and its pivotal role in determining potential treatment outcomes. Patients should be stratified accordingly, when the treatment algorithms are considered. Furthermore, a multi-level diagnostic approach, including performance of individualised molecular profiling (i.e. with appropriate blood tests), is essential to achieve predictable outcomes in well stratified patients/patient cohorts.

## Conclusions

The matrix metalloproteinases MMP-2 and MMP-9 are well known prognostic factors that, when elevated, are indicators of poor outcomes for oncologic patients. The finding of increased MMP activity, as short- and long-term effects in the patient group with breast cancer with liver metastases treated with SIRT, has profound implications if it can be shown that this response was provoked predominantly by the SIRT. If SIRT is to be employed in these patients, it will be important to perform follow-up investigations in this patient cohort to evaluate complementary molecular pathways that may be co-responsible for the extended tumour growth and for the accelerated metastatic activity observed in these patients. After the overall picture of the molecular events is completed, an important conclusion will be possible to make, namely, whether SIRT might be potentially considered as inappropriate for treatment of some, if not all, hepatic metastases from breast cancer.

**Fig. 4:**
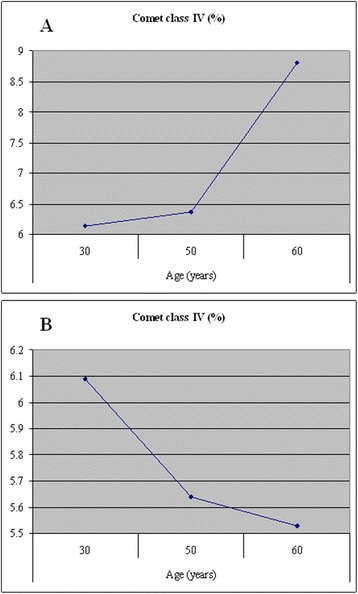
“Comet Assay” analysis in breast cancer patients. The DNA analysis has been performed ex vivo using circulating leukocytes of **a**. control group *versus* **b**. breast cancer patients. Apoptotic rates (class VI) have been correlated with decades of life in the pools of comparison. It is evident that the apoptotic rates normally tend to increase in the 5th and 6th decade of life. In contrast, the breast cancer patients demonstrate much more heterogeneous image and general tendency to decrease after the 3rd life decade. These results have been published earlier [25]

This may be the case, if any stratified patient cohorts with primary breast cancer are identifiable as more resistant to irradiation compared to baseline. Some indications for this kind of evaluation have been published earlier by the authors [25]. This is to provide the main message from the previous study supporting current results and recommendations. It has been clearly demonstrated (see Fig. 4a) that the controls tend to increasing apoptotic rates towards progressing age. In contrast, Fig. 4b shows an inverse tendency in breast cancer patients, with apoptotic rates generally decreasing after the 3rd decade of life. Therefore, a conclusion has been made that at least some of the breast cancer subgroups are more resistant against apoptosis and, consequently may be more resistant against irradiation, if compared to baseline. Radiation resistance is the parameter crucial for corresponding patient stratification and should be thoroughly investigated in patient cohorts with different liver cancer subtypes, in order to make more optimal decisions for individualised therapy approaches.

## Abbreviations

BCMD, breast cancer metastatic disease; CAFs, cancer-associated fibroblasts; CNAPs, circulating nucleic acids in plasma; CT, X-ray computed tomography; ELISA, enzyme linked immunosorbent Assay; ET-1, endothelin-1; HCC, hepatocellular carcinoma; MMP-2, matrix metalloproteinase 2; MMP-9, matrix metalloproteinase 9; PET, positron emission tomography; proET-1, C-terminal proendothelin-1; SIRT, selective internal radiation therapy; TACE, transarterial chemoembolisation; TIMP, tissue inhibitor of metalloproteinases; VEGF, vascular endothelial growth factor
